# Chiral deaza-coelenterazine analogs for probing a substrate-binding site in the Ca^2+^-binding photoprotein aequorin

**DOI:** 10.1371/journal.pone.0251743

**Published:** 2021-06-11

**Authors:** Satoshi Inouye, Yuto Sumida, Yuri Tomabechi, Jumpei Taguchi, Mikako Shirouzu, Takamitsu Hosoya

**Affiliations:** 1 Yokohama Research Center, JNC Co., Yokohama, Japan; 2 Laboratory for Chemical Biology, RIKEN Center for Biosystems Dynamics Research (BDR), Kobe, Japan; 3 Laboratory for Protein Functional and Structural Biology, RIKEN Center for Biosystems Dynamics Research (BDR), Yokohama, Japan; 4 Laboratory of Chemical Bioscience, Institute of Biomaterials and Bioengineering, Tokyo Medical and Dental University, Tokyo, Japan; Indian Institute of Technology Kharagpur, INDIA

## Abstract

The Ca^2+^-binding photoprotein aequorin is a complex of apoAequorin (apoprotein) and (*S*)-2-peroxycoelenterazine. Aequorin can be regenerated by the incubation of apoAequorin with coelenterazine and molecular oxygen (O_2_). In this study, to investigate the molecular recognition of apoAequorin for coelenterazine using chemical probes, the chiral deaza-analogs of (*S*)- and (*R*)-deaza-CTZ (daCTZ) for coelenterazine and of (*S*)-2- and (*R*)-2-hydroxymethyl-deaza-CTZ (HM-daCTZ) for 2-peroxycoelenterazine were efficiently prepared by the improvement method. The chiral deaza-analogs of (*S*)-daCTZ and (*S*)-HM-daCTZ selectively inhibited the regeneration step to aequorin by binding the catalytic site of coelenterazine in the apoAequorin molecule. The crystal structures of the apoAequorin complexes with (*S*)-daCTZ and (*S*)-HM-daCTZ were determined, suggesting that the hydroxy moiety at the C6-hydroxyphenyl group and the carbonyl moiety of the imidazopyrazinone ring in coelenterazine are essential to bind the apoAequorin molecule through hydrogen bonding. Therefore, the chiral deaza-analogs of coelenterazine can be used as a probe to study the interaction between coelenterazine and the related proteins including photoprotein, luciferase, and coelenterazine-binding protein.

## Introduction

**Fig 1:**
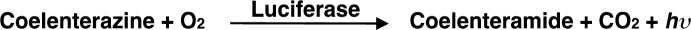
The luminescence reaction of coelenterazine and molecular oxygen catalyzed by luciferase.

Coelenterazine with an imidazopyrazinone structure (3,7-dihydroimizazopyrazin-3-one) is widely distributed among luminous and non-luminous marine organisms including coelenterates (jellyfish and colonial cnidarian), arthropods (copepod, ostracod, and crustacean), squids, and fishes [1]. In the bioluminescent organisms, coelenterazine is used as a luciferin (a substrate) for the luminescence reaction with a luciferase (an enzyme). The oxidation of coelenterazine with O_2_ by luciferase results in light emission, accompanied by the production of coelenteramide and CO_2_ according to the following reaction scheme: (Fig 1)

In the oxidation process of coelenterazine to coelenteramide by luciferase, the peroxy-intermediates of coelenterazine might be involved [2–4].

**Fig 2:**
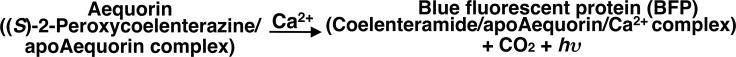
The luminescence reaction of aequorin triggered by Ca^2+^ and the formation of blue fluorescent protein (BFP).

**Fig 3:**
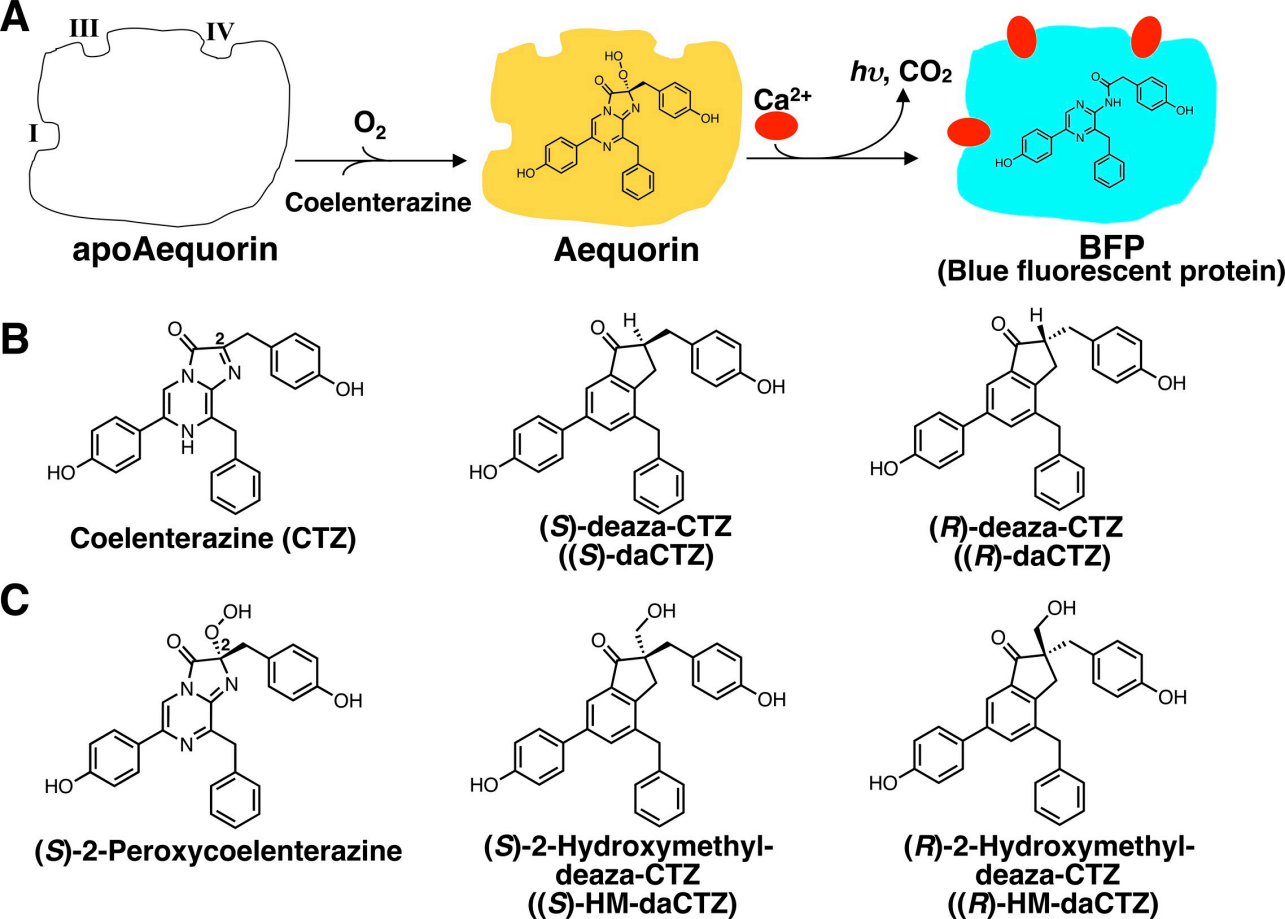
Ca^2+^-triggered luminescence reaction of aequorin, regenerated from apoAequorin and coelenterazine. A. Regeneration of aequorin from apoAequorin and coelenterazine, followed by the calcium triggered-luminescence reaction. B. Structures of coelenterazine (CTZ) and its chiral deaza-analogs of (*S*)- and (*R*)-deaza-CTZ (daCTZ). C. Structures of (*S*)-2-peroxycoelenterazine and the chiral deaza-analogs of (*S*)-2- and (*R*)-2-hydroxymethyl-deaza-CTZ (HM-daCTZ).

Coelenterazine also serves as a light-emitting source of the Ca^2+^-binding photoproteins (a complex of (*S*)-2-peroxycoelenterazine and an apoprotein) including aequorin (PDB ID: 1EJ3) [5], obelin (PDB ID: 1QV1) [6] clytin (PDB ID: 3KPX) [7], and mitrocomin (PDB ID: 4NQG) [8], which were identified in the coelenterates. The well-characterized Ca^2+^-binding photoprotein aequorin emits light by an intramolecular reaction with a trace amount of Ca^2+^ (>10^−7^ M), yielding the blue fluorescent protein [BFP, a complex of coelenteramide and apoprotein (apoAequorin)] and CO_2_, as shown in the following reaction scheme [9–11] (Fig 2):

apoAequorin, which consists of 189 amino acid residues [12], can be regenerated to aequorin by incubation with coelenterazine and O_2_, both *in vivo* [13–15] and *in vitro* [16, 17] (Fig 3).

In 2001, Nakamura *et al*. described the deaza-analogs of coelenterazine and 2-peroxycoelenterazine as inhibitors to investigate the stereo-specificity of the oxidation process of coelenterazine in the coelenterazine-utilizing luciferase reaction (Fig 3B) [3, 18]. They demonstrated that *Renilla* luciferase, which is one of the coelenterazine-utilizing luciferases in the coelenterates, exhibited the luminescence kinetics of competitive inhibition with the deaza-analogs of (*R*)-deaza-CTZ ((*R*)-daCTZ) and (*R*)-2-hydroxymethyl-deaza-CTZ ((*R*)-HM-daCTZ) (Fig 3B and 3C). As a result, the oxidation process with *Renilla* luciferase occurs stereo-specifically through the peroxide intermediate of (*R*)-2*-*peroxycoelenterazine. On the other hand, the structure of the Ca^2+^-binding photoprotein aequorin was determined by X-ray structural analysis, and the results indicate that (*S*)-2*-*peroxycoelenterazine as a light-emitting source is stabilized in the apoAequorin molecule [5]. Therefore, the oxidation process of coelenterazine with O_2_ in the coelenterazine-utilizing luciferases and the photoproteins involved in the stereospecific oxygenation process, and the chiral deaza-analogs could be used as a probe to investigate the mechanisms of the peroxy-intermediate oxidation of coelenterazine, and also the binding environments of coelenterazine in luciferases and photoproteins.

In this study, an improved synthetic route to prepare deaza-analogs is described and their application to characterize the oxidation process of coelenterazine in the regeneration step to aequorin from apoAequorin, based on the crystal structure analyses of the complexes of apoAequorin and the chiral deaza-analogs are demonstrated.

## Materials and methods

### Materials

Recombinant histidine-tagged apoAequorin (198 amino acids, Mw = 22,512.1) was expressed into the periplasmic space of *E*. *coli* cells using a piP-His-HE expression vector and purified, as previously described [19]. Coelenteramide (CTMD) was chemically synthesized according to a previously described procedure [19]. The following chemicals were obtained from commercial sources: coelenterazine (JNC. Co., Tokyo, Japan); 2-mercaptoethanol, dithiothreitol (DTT), dimethyl sulfoxide (DMSO), ethylenediaminetetraacetic acid disodium salt (EDTA**·**2Na), and imidazole (Wako Pure Chemicals, Osaka, Japan); Butyl-Sepharose FF (GE Healthcare, Piscataway, NJ, USA). All other chemicals were of the highest commercial grade available.

### Luminescence activity determination

**a) Luminescence activity of aequorin.** The regeneration to aequorin was performed by incubating recombinant His-apoAequorin with coelenterazine in 30 mM Tris-HCl (pH 7.6)–10 mM EDTA at 4°C in the presence of 0.1% 2-mercaptoethanol. A portion of the regenerated mixture (1–5 μL) was placed in a polystyrene tube (12 mm × 75 mm, Cat. No. TYS-210, BM Equipment Co.) and the luminescence activity was measured in 0.1-s intervals for 60 s by injecting 100 μL of 50 mM CaCl_2_ in 50 mM Tris-HCl (pH 7.6) at room temperature (23–25°C) using an ATTO (Tokyo, Japan) model AB2200 luminometer (ver. 2.07, rev4.21) with or without a 0.23% neutral density filter at 550 nm. The maximum intensity of luminescence (*I*_max_) was shown as relative light units (rlu).

**b) Luciferase-like activity of apoAequorin.** The luciferase-like activities of apoAequorin and its complexes were determined by the addition of coelenterazine (1 μg/μL, dissolved in ethanol) to 100 μL of the reaction mixture, and the luminescence activity was determined using a luminometer in 0.1-s intervals for 60 s.

### Chemical syntheses of the deaza-coelenterazine analogs of deaza-CTZ (daCTZ) and 2-hydroxymethyl deaza-CTZ (HM-daCTZ)

**Fig 4:**
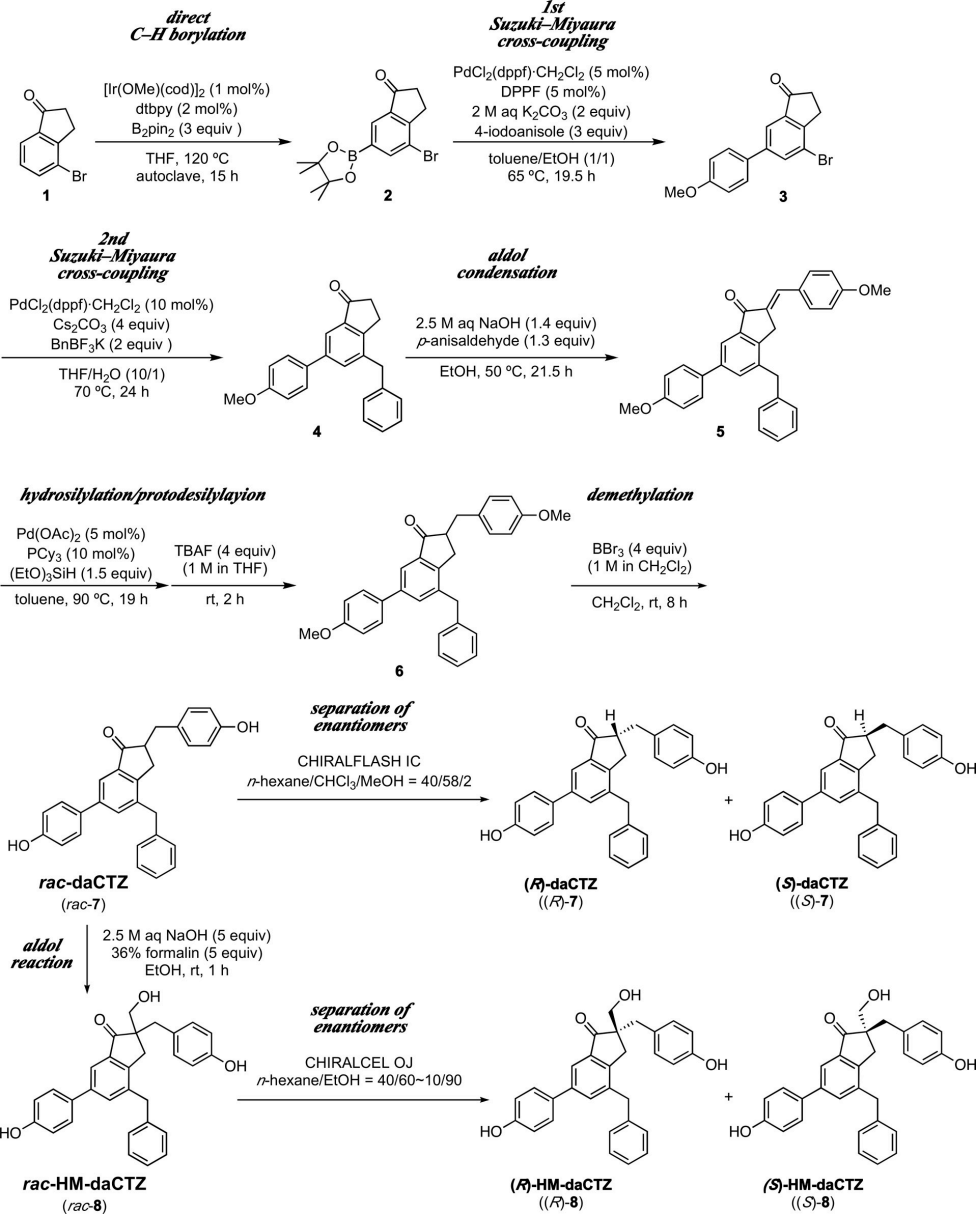
Synthetic route of the racemic deaza-CTZ (daCTZ) compounds, (*R*)- and (*S*)-daCTZ, and hydroxymethyl-deaza-CTZ (HM-daCTZ), (*R*)-2- and (*S*)-2-HM-daCTZ, and each enantiomer separation.

As shown in Fig 4, the enantiomers of (*R*)-daCTZ ((*R*)-**7**) and (*S*)-daCTZ ((*S*)-**7**), and of (*R*)-HM-daCTZ ((*R*)-**8**) and (*S*)-HM-daCTZ ((*S*)-**8**) were prepared according to a method previously reported [3, 18] with several modifications using modern synthetic methods (S1 Methods). Briefly, the racemic compounds of daCTZ (*rac*-**7**) for (*R*)- and (*S*)-daCTZ and of HM-daCTZ (*rac*-**8**) for (*R*)- and (*S*)-HM-daCTZ were synthesized and each enantiomer was isolated using HPLC with a preparative chiral column (S1 Fig in S1 File).

### Preparation of the apoAequorin/(*S*)-HM-daCTZ complex

The purified histidine-tagged apoAequorin (59.0 mg, 2.6 μmol) and (*S*)-HM-daCTZ (1.2 mg dissolved in 0.5 mL of DMSO, 2.7 μmol) was suspended in 50 mL of 50 mM Tris-HCl (pH 7.6)–10 mM EDTA containing 10 mM DTT. The mixture was incubated at 4°C for 20 h and then adjusted to the final concentration of 2 M (NH_4_)_2_SO_4_ by adding 50 mL of 4 M (NH_4_)_2_SO_4_ dissolved in TE [10 mM Tris-HCl (pH 7.6)–2 mM EDTA] and was applied on a Butyl-Sepharose 4 Fast Flow column (ø1.5 × 5.0 cm) equilibrated with 2 M (NH_4_)_2_SO_4_ in TE at room temperature. After washing the column with 100 mL of 2 M (NH_4_)_2_SO_4_ in TE, the adsorbed apoAequorin/(*S*)-HM-daCTZ complex was eluted with 1.2 M (NH_4_)_2_SO_4_ in TE. The fractions showing the absorbance peak ratio at 330 nm to 280 nm over 0.024 were collected. The protein yield of the apoAequorin/(*S*)-HM-daCTZ complex was 45.1 mg (76.4%).

### Preparation of the apoAequorin/(*S*)-daCTZ complex

The procedures for preparing the apoAequorin/(*S*)-daCTZ complex were essentially the same as that of the apoAequorin/(*S*)-HM-daCTZ complex, except for the elution conditions of (NH_4_)_2_SO_4_ from the Butyl-Sepharose column. The reaction mixture was composed of histidine-tagged apoAequorin (53.6 mg, 2.4 μmol) and (*S*)-daCTZ (1.1 mg dissolved in 0.5 mL of DMSO, 2.6 μmol) in 50 mL of TE containing 10 mM DTT. After incubation at 4°C for 20 h, 50 mL of 4 M (NH_4_)_2_SO_4_ in TE was added to the mixture to a final concentration of 2 M (NH_4_)_2_SO_4_. The solution became cloudy and was immediately applied on a Butyl-Sepharose 4 Fast Flow column (ø1.5 × 7.0 cm). After washing stepwise with 40 mL of 2 M, 1.2 M, 0.8 M, and 0.4 M (NH_4_)_2_SO_4_ in TE, the (*S*)-daCTZ/apoAequorin complex with light-yellow color was eluted with 0.4 M (NH_4_)_2_SO_4_ in TE, and the fractions showing the absorbance peak ratio at 280 nm to 330 nm over 0.031 were collected. A fraction of the protein adsorbed on the gel top was not eluted with TE and might be denatured. The protein yield was 14. 1 mg (26.3%).

### Absorption spectrum measurements

The absorption spectra were measured using a Jasco (Tokyo, Japan) V-560 spectrophotometer (bandwidth 0.5 nm; response, medium; scan speed, 100 nm/min) at 22–25°C using a quartz cuvette (10-mm light path).

### Protein analysis

The protein concentration was determined by the dye-binding method using a commercially available kit (Bio-Rad, Richmond, CA, USA) and bovine serum albumin as a standard (Pierce, Rockford, IL, USA).

### Crystallization, data collection, and structure determination

The crystallization of the apoAequorin/(*S*)-HM-daCTZ and apoAequorin/(*S*)-daCTZ complexes was performed by the hanging drop vapor diffusion method, as previously described [20]. Briefly, the solutions of apoAequorin complexes with HM-daCTZ and daCTZ were concentrated to the protein concentrations of 18.8 mg/mL and 16.6 mg/mL, respectively, using an Amicon Ultra centrifugal filter unit (MWCO 10,000). The crystals grew in a mixture of 1 μL of the complex solution and 1 μL of the precipitant solution at 20°C. The precipitant solutions for the apoAequorin/(*S*)-HM-daCTZ complex and apoAequorin/(*S*)-daCTZ complex were 2 M (NH_4_)_2_SO_4_ in 0.1 M Tris-HCl (pH 8.5) and 1.9 M (NH_4_)_2_SO_4_ in 0.2 M K_2_HPO_4_, respectively. After a few days of incubation for equilibration against the precipitant solution, single light-yellow crystals with dimensions of 150 μm × 50 μm × 50 μm for the apoAequorin/(*S*)-HM-daCTZ and 180 μm × 50 μm × 50 μm for the apoAequorin/(*S*)-daCTZ complex were obtained (S2 Fig in S1 File). The crystals were cryoprotected in a reservoir solution supplemented with 20% (v/v) glycerol before flash-cooling in liquid nitrogen. An X-ray diffraction dataset was collected to 2.2 Å for the apoAequorin/(*S*)-HM-daCTZ complex and 2.1 Å for the apoAequorin/(*S*)-daCTZ at a wavelength of 1.0 Å on beamline BL26B2 at SPring-8 [21]. The diffraction data were processed using the XDS programs [22], and the structure was solved by molecular replacement using the Phaser program [23] from the PHENIX programs [24], with the aequorin coordinates (PDB ID: 1EJ3) as the search model [5]. The structural model was built into the electron density map using Coot [25] and refined using the PHENIX program [24]. Structure figures were prepared using the PyMOL Molecular Graphics System, version 2.07.

## Results and discussion

### Improved synthesis of deaza-coelenterazine analogs as inhibitors

Previously, Nakamura *et al*. reported the synthesis of the racemic deaza-analogs for coelenterazine and 2-peroxycoelenterazine, and each enantiomer was separated using a chiral column [3, 18] (Fig 3B). In this study, we improved the synthetic methods to produce the racemic compounds of (*R*)-daCTZ ((*R*)-**7**) and (*S*)-daCTZ ((*S*)-**7**) (*rac*-daCTZ, *rac*-**7**), and of (*R*)-HM-daCTZ ((*R*)-**8**) and (*S*)-HM-daCTZ ((*S*)-**8**) (*rac*-HM-daCTZ, *rac*-**8**) (Fig 4). In brief, the synthesis of the key intermediate compound, 4-benzyl-6-(4-methoxyphenyl)-1-indanone (**4**), was achieved from commercial 4-bromo-1-indanone (**1**) in three steps, involving iridium-catalyzed regioselective C–H borylation and consecutive palladium-catalyzed cross-coupling reactions with 4-iodoanisole and potassium benzyltrifluoroborate. The overall yield of **4** from **1** (63%) was largely improved in comparison with that of the previous method in which compound **4** was prepared from 4-bromo-*N*,*N*-diethylbenzamide in 32% overall yield through six steps [3, 18]. The formal hydrogenation of the olefin moiety of enone **5** by a palladium-catalyzed hydrosilylation/fluoride-mediated protodesilylation sequence achieved a better result than the reported platinum-catalyzed hydrogenation for the synthesis of compound **6** [18]. These improvements enabled the synthesis of deaza-coelenterazine analogs on a larger scale than that previously reported [3, 18], and facilitated the revision of the characterization data of the synthesized compounds including the intermediates (S1 Methods). In addition, other deaza-type inhibitors of coelenterazine analogs are easy to be synthesized by the new synthetic route using compound **2** as the common intermediate.

After separation of the racemic mixture by HPLC using a column packed with a chiral stationary phase, the optical purities for (*S*)-daCTZ and (*R*)-daCTZ were 99.6% ee and 99.2% ee, respectively. For (*S*)-HM-daCTZ and (*R*)-HM-daCTZ, slightly lower optical purities of 97.0% ee and 97.3% ee, respectively, were observed, which might be explained by the racemization of the separated isomers via the retro-aldol reaction.

### Selective inhibition of aequorin regeneration from apoAequorin and coelenterazine in the presence of deaza-analogs

**Fig 5:**
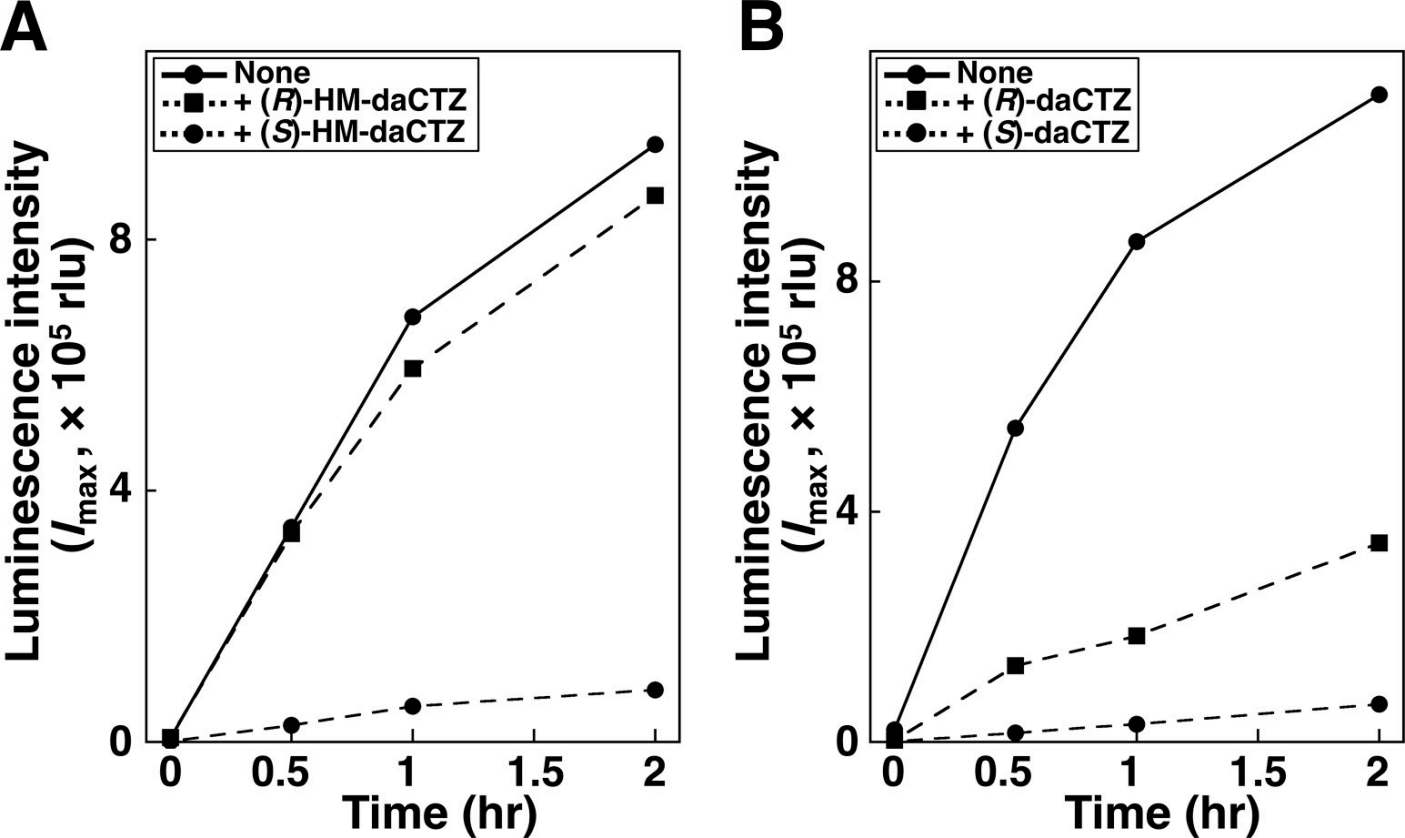
Inhibition of aequorin regeneration from apoAequorin and coelenterazine in the presence of chiral deaza-coelenterazine analog. A. Time course of aequorin regeneration from apoAequorin and coelenterazine in the presence of (*R*)-2- and (*S*)-2-hydroxymethyl deaza-CTZ (HM-daCTZ). B. Time course of aequorin regeneration from apoAequorin and coelenterazine in the presence of (*S*)- and (*R*)-deaza-CTZ (daCTZ). The reaction conditions are described in Table 1.

Aequorin can be regenerated from apoAequorin by incubation with coelenterazine and O_2_ in the presence of reducing reagents at 4°C in a few hours (Fig 3A). The inhibitory effects on aequorin generation were examined by incubating with the chiral deaza-analogs of (*R*)-daCTZ and (*S*)-daCTZ for coelenterazine and (*R*)-HM-daCTZ and (*S*)-HM-daCTZ for 2-peroxycoelenterazine (Fig 3B and 3C). Because the chirality at the C2-peroxy moiety of 2-peroxycoelenterazine in aequorin is the *S* form [5], (*S*)-HM-daCTZ showed strong inhibition on the regeneration to aequorin, but not (*R*)-HM-daCTZ (Fig 5A, Table 1). Moreover, the deaza-analog of (*R*)-daCTZ and (*S*)-daCTZ for coelenterazine inhibited the aequorin regeneration and the inhibition with (*S*)-daCTZ was stronger than that with (*R*)-daCTZ (Fig 5B).

**Table 1:** Inhibition of aequorin regeneration form apoAequorin and coelenterazine in the presence of chiral deaza-analogs.

Incubation time (h)	Relative luminescence activity (*I*_max_, %) ^a^				
	Coelenterazine (CTZ)	+ (*S*)- HM-daCTZ	+ (*R*)- HM-daCTZ	+ (*S*)- daCTZ	+ (*R*)- daCTZ
2	100	8.7	92.1	5.8	33.4
24	100	32.6	97.8	31.0	64.3

^a^ Regeneration mixture contains 1 μg recombinant His-apoAequorin (0.044 nmol, 8.9 × 10^−8^ M) in 500 μL of 30 mM Tris-HCl (pH 7.6)–10 mM EDTA with 1 μL of 2-mercaptoethanol. After the addition of 1 μg/μL coelenterazine (2.4 nmol, 4.7 × 10^−6^ M) and deaza-analogs [2 μg/μL daCTZ (4.7 nmol, 9.5 × 10^−6^ M) or 1 μg/1 μL HM-daCTZ (2.4 nmol, 4.7 × 10^−6^ M)] dissolved in ethanol to the regeneration mixture, the mixture was incubated at 4°C. The luminescence activity of 5 μL was determined by injecting 100 μL of 50 mM CaCl_2_ in 50 mM Tris-HCl (pH 7.6) using a luminometer.

Unexpectedly, after incubation with coelenterazine for 24 h, the regeneration efficiency to aequorin in the presence of (*S*)-HM-daCTZ, (*S*)-daCTZ, and (*R*)-daCTZ increased slowly to 32.6%, 31.0%, and 64.8%, respectively (Table 1). Therefore, the binding affinity of coelenterazine to apoAequorin was higher than that of (*S*)-HM-daCTZ, (*S*)-daCTZ, and (*R*)-daCTZ, and the replacement of these inhibitors with coelenterazine occurred spontaneously. These results suggest that the regeneration process to aequorin is a stereospecific reaction in which coelenterazine binds to apoAequorin, followed by oxygenation with O_2_.

### Preparation of the complexes of apoAequorin and deaza-analogs

**Fig 6:**
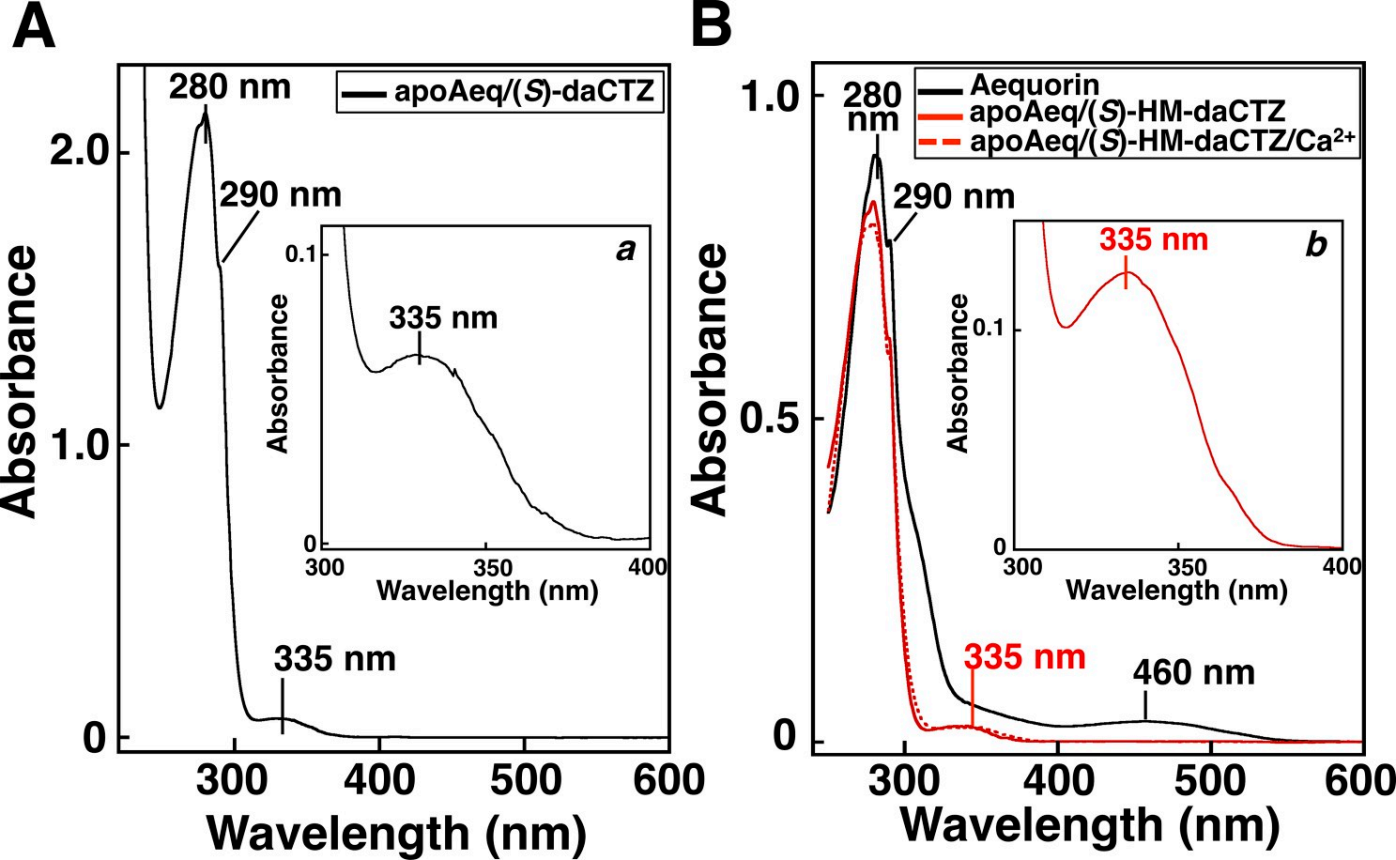
Absorption spectra of apoAequorin/(*S*)-HM-daCTZ complex, apoAequorin/(*S*)-daCTZ complex, and recombinant aequorin. A. Absorption spectrum of the apoAequorin/(*S*)-HM-daCTZ complex eluted from the Butyl-Sepharose column. Protein concentration is 0.80 mg/mL in 0.4 M (NH_4_)_2_SO_4_ of 10 mM Tris-HCl (pH 7.6)–2 mM EDTA. B. Absorption spectra of the apoAequorin/(*S*)-daCTZ complex and aequorin eluted from the Butyl-Sepharose column. Protein concentration of apoAequorin/(*S*)-daCTZ complex and recombinant aequorin are 0.29 mg/mL and 0.32 mg/mL, respectively, in 1.2 M (NH_4_)_2_SO_4_ of 10 mM Tris-HCl (pH 7.6)–2 mM EDTA. Protein concentration in ***b*** is 1.36 mg/mL. The final concentration of Ca^2+^ in apoAequorin/(*S*)-daCTZ complex is 50 mM of CaCl_2_.

The strong inhibition of (*S*)-daCTZ and (*S*)-HM-daCTZ on aequorin regeneration indicate that the deaza-analogs can generate a stable complex with apoAequorin. The complexes of apoAequorin and the deaza-analogs were prepared by incubating apoAequorin with (*S*)-daCTZ and (*S*)-HM-daCTZ, and the deaza-analog complexes were separated from the unbound apoAequorin and free deaza-analogs by hydrophobic chromatography using a Butyl-Sepharose column. The absorption spectra of the purified apoAequorin complexes with (*S*)-daCTZ and (*S*)-HM-daCTZ were determined and the absorbance peak displayed at 335 nm derived from the indanone structures of (*S*)-daCTZ and (*S*)-HM-daCTZ (Fig 6). The incorporation ratio of the deaza-analog to apoAequorin was estimated to be over 95%, based on the molar absorbance coefficient of the racemic mixture of (*S*)- and (*R*)-daCTZ (*rac*-daCTZ, *rac*-**7**) at 330 nm (ε = 2,200 M^–1^cm^–1^ in ethanol). The absorption spectrum of the apoAequorin/(*S*)-HM-daCTZ complex was distinct from that of native aequorin, which consisted of (*S*)-2-peroxycoelenterazine and apoAequorin. Furthermore, the addition of Ca^2+^ to the apoAequorin/(*S*)-HM-daCTZ complex did not affect the absorption spectrum significantly (Fig 6B).

### Characterization of the apoAequorin/(*S*)-HM-daCTZ complex

**Fig 7:**
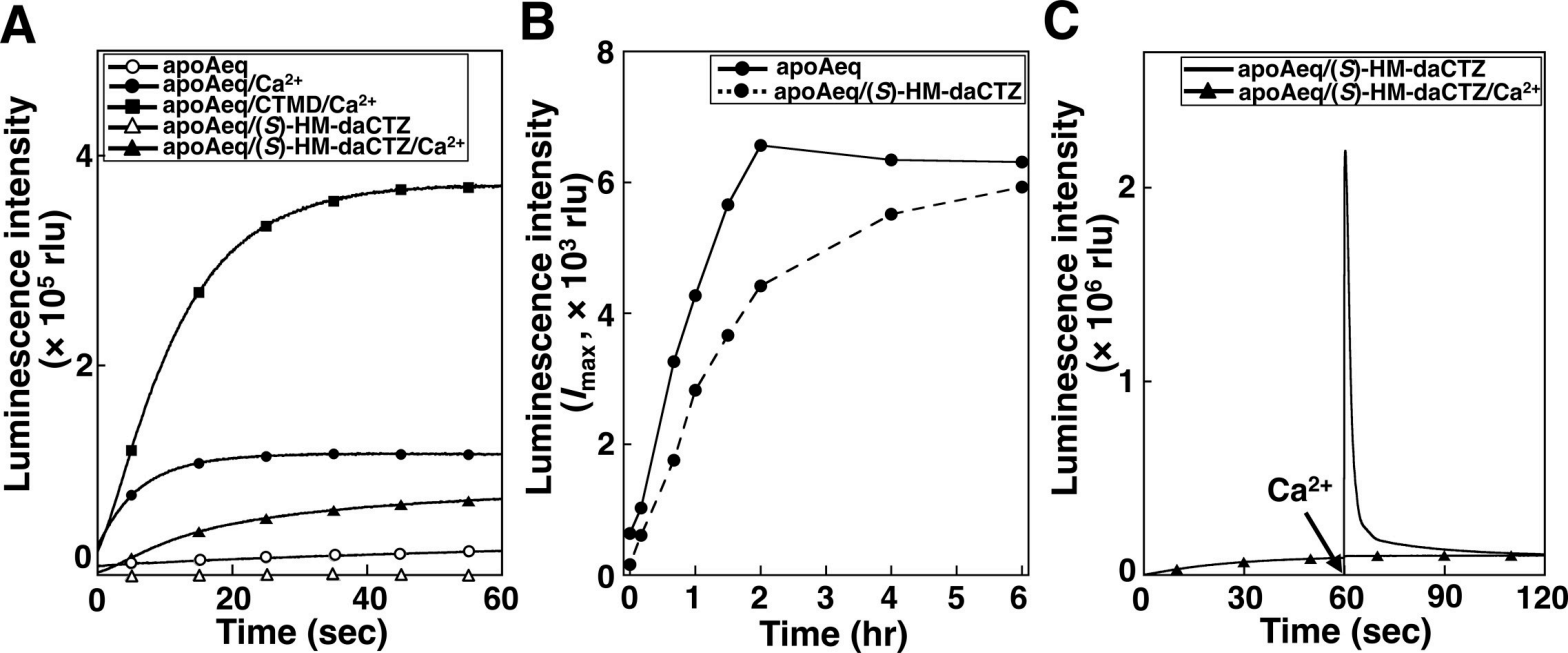
Luminescence properties of the apoAequorin/(*S*)-HM-daCTZ complex, by comparison with apoAequorin. A. Comparison of the luciferase-like luminescence reaction of the apoAequorin/(*S*)-HM-daCTZ/Ca^2+^ complex with the complex of apoAequorin, coelenteramide (CTMD), and Ca^2+^. The reaction mixture contains 1 μg of apoAequorin in 100 μL of Tris-HCl (pH 7.6) in the presence of 10 mM CaCl_2_, 1 μg/μL of CTMD, and/or 1 μg/μL of (*S*)-HM-daCTZ. The luminescence reaction was started by the addition of 1 μg/μL of coelenterazine at 22°C and the luminescence activity was determined using a luminometer. B. Time course of aequorin regeneration from the apoAequorin/(*S*)-HM-daCTZ complex with coelenterazine. The regeneration conditions are the same as in Fig 5A. The luminescence activity of 5 μL in the mixture was determined using a luminometer with a 0.23% neutral density filter. The bold line and dashed line indicate apoAequorin and the apoAequorin/(*S*)-HM-daCTZ complex, respectively. C. Ca^2+^-triggered luminescence reaction of aequorin regenerated from the apoAequorin/(*S*)-HM-daCTZ complex and coelenterazine. The mixture includes 1 μg of apoAequorin or the apoAequorin/(*S*)-HM-daCTZ complex in 1 mL of 30 mM Tris-HCl (pH 7.6)–10 mM EDTA containing 1 μg/μL of coelenterazine and 1 μL of 2-mercaptoethanol and incubated at 4°C. The luminescence activity of 1 μL of regenerated aequorin was determined by injection of 100 μL of 50 mM CaCl_2_ using a luminometer.

Previously, we reported that apoAequorin (E), the apoAequorin/Ca^2+^ complex (E/Ca^2+^), and the apoAequorin/coelenteramide/Ca^2+^ complex (E/P/Ca^2+^, BFP) could catalyze the oxidation of coelenterazine to produce a continuous weak luminescence [10, 11]. As expected, the apoAequorin/(*S*)-HM-daCTZ/Ca^2+^ complex (E/I/Ca^2+^) showed a continuous luminescence with coelenterazine, similar to that of E/P/Ca^2+^ and E/Ca^2+^. The *I*_max_ ratio (%) for E/P/Ca^2+^, E/Ca^2+^, E/I/Ca^2+^, E, and E/I were 100, 30.3, 19.3, 6.8, and 0.01, respectively (Fig 7A).

The replacement of (*S*)-HM-daCTZ in the apoAequorin/(*S*)-HM-daCTZ complex with coelenterazine was confirmed by incubating the isolated apoAequorin/(*S*)-HM-daCTZ complex with coelenterazine and then the Ca^2+^-triggered luminescence activity of the regenerated aequorin was determined. As a result, the apoAequorin/(*S*)-HM-daCTZ complex was converted to aequorin (Fig 7B), exhibiting the Ca^2+^-triggered flash luminescence (Fig 7C). Similarly, the regeneration to aequorin from the apoAequorin/(*S*)-daCTZ complex by incubation with coelenterazine was performed, and the results revealed that the deaza-analog binds non-covalently to apoAequorin, enabling the replacement with coelenterazine to convert active aequorin.

### Structural determination of the apoAequorin/(*S*)-daCTZ and apoAequorin/(*S*)-HM-daCTZ complexes

In the regeneration process to aequorin from apoAequorin by incubation with coelenterazine and O_2_, the recognition process of apoAequorin to bind coelenterazine and the following oxygenation steps were unclear. As the deaza-analogs of (*S*)-daCTZ and (*S*)-HM-daCTZ were regarded as coelenterazine and (*S*)-2-peroxycoelenterazine, respectively, (*S*)-daCTZ could be used as a probe to determine the recognition residues of apoAequorin and (*S*)-HM-daCTZ could be applied to confirm the binding environment of (*S*)-2-peroxycoelenterazine in apoAequorin, as previously reported [5].

The crystal structures of the complexes of (*S*)-HM-daCTZ and (*S*)-daCTZ with apoAequorin were determined at 2.1 Å resolution (PDB ID: 7EG3 and PDB ID: 7EG2, respectively). The statistical values of data collection and structure refinement are summarized in Table 2.

**Table 2:** Statistics of data collection and structure refinement.

	(*S*)-HM-daCTZ	(*S*)-daCTZ
***Data collection and processing***		
Beamline	BL26B2	BL26B2
Space group	*P*1	*P*1
Unit-cell parameter		
*a*, *b*, *c* (Å)	91.31, 98.14, 121.48	90.80, 98.02, 121.42
*α*, *β*, *γ* (Å)	77.47, 73.47, 75.16	77.63, 73.23,75.22
Wavelength (Å)	1.000	1.000
Resolution range (Å)	50–2.22 (2–2.22)	50–2.09 (2.23–2.09)
Redundancy	1.99 (1.98)	3.90 (3.82)
Completeness (%) ^a^	95.5 (90.0)	97.35 (93.5)
*R*_sym_^b^ (%) ^a^	13.0 (94.6)	9.4 (50.7)
*I/σ* (*I*) ^a^	7.75 (1.37)	11.64 (3.28)
No. monomers/asymmetric unit	16	16
***Model refinement***		
No. of reflections	183193	219443
No. of protein atoms	24426	24376
No. of water molecules	1524	1435
*R*_work_/*R*_free_ ^c^ (%)	20.46/24.53	21.77/24.46
r.m.s.d. for bond length (Å)	0.002	0.002
r.m.s.d. for bond angles (˚)	0.414	0.509
***Residues in the Ramachandran plot***		
Favored region (%)	99.14	99.30
Allowed regions (%)	0.86	0.70
PDB entry	7EG3	7EG2

^a^ Statistics for the highest resolution shell are given in parentheses.

^b^ *R*_sym_ = (∑_*h*_∑_*i*_|*I*_*hi*_–‹*I*_*h*_›|/∑_*h*_∑_*i*_|*I*_*hi*_|) where *h* indicates unique reflection indices and *i* indicates symmetry equivalent indices.

^c^ *R*_work_ = ∑|*F*_obs_–*F*_calc_|/∑*F*_obs_ for all reflections and *R*_free_ was calculated using randomly selected reflections (6%).

**Fig 8:**
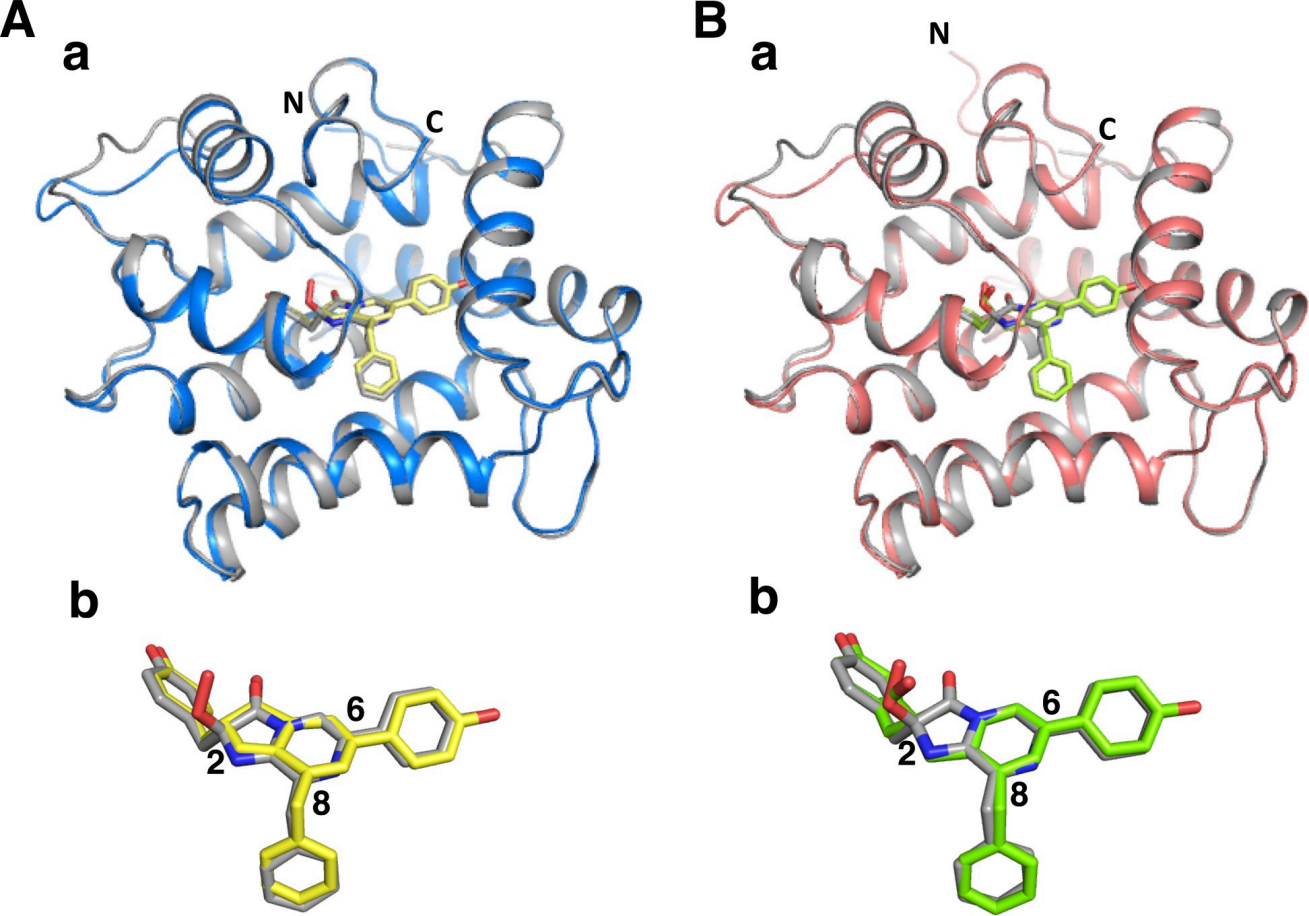
Crystal structures of the apoAequorin complex with (*S*)-daCTZ and (*S*)-HM-daCTZ. A. a) Superposition of native aequorin (A form, gray color) and the apoAequorin/(*S*)-daCTZ complex (A form, blue color). b) Superposition of (*S*)-2-peroxycoelenterazine (gray color) and (*S*)-daCTZ (yellow color). B. a) Superposition of native aequorin (A form, gray color) and the apoAequorin/(*S*)-HM-daCTZ complex (A form, red color). b) Superposition of (*S*)-2-peroxycoelenterazine (gray color) and (*S*)-HM-daCTZ (green color). The labeled N and C in the structures indicate the amino and carboxyl terminus, respectively. The numbers 2, 6, and 8 indicate the positions of C2, C6, and C8 in the imidazopyrazinone ring of coelenterazine, respectively.

**Fig 9:**
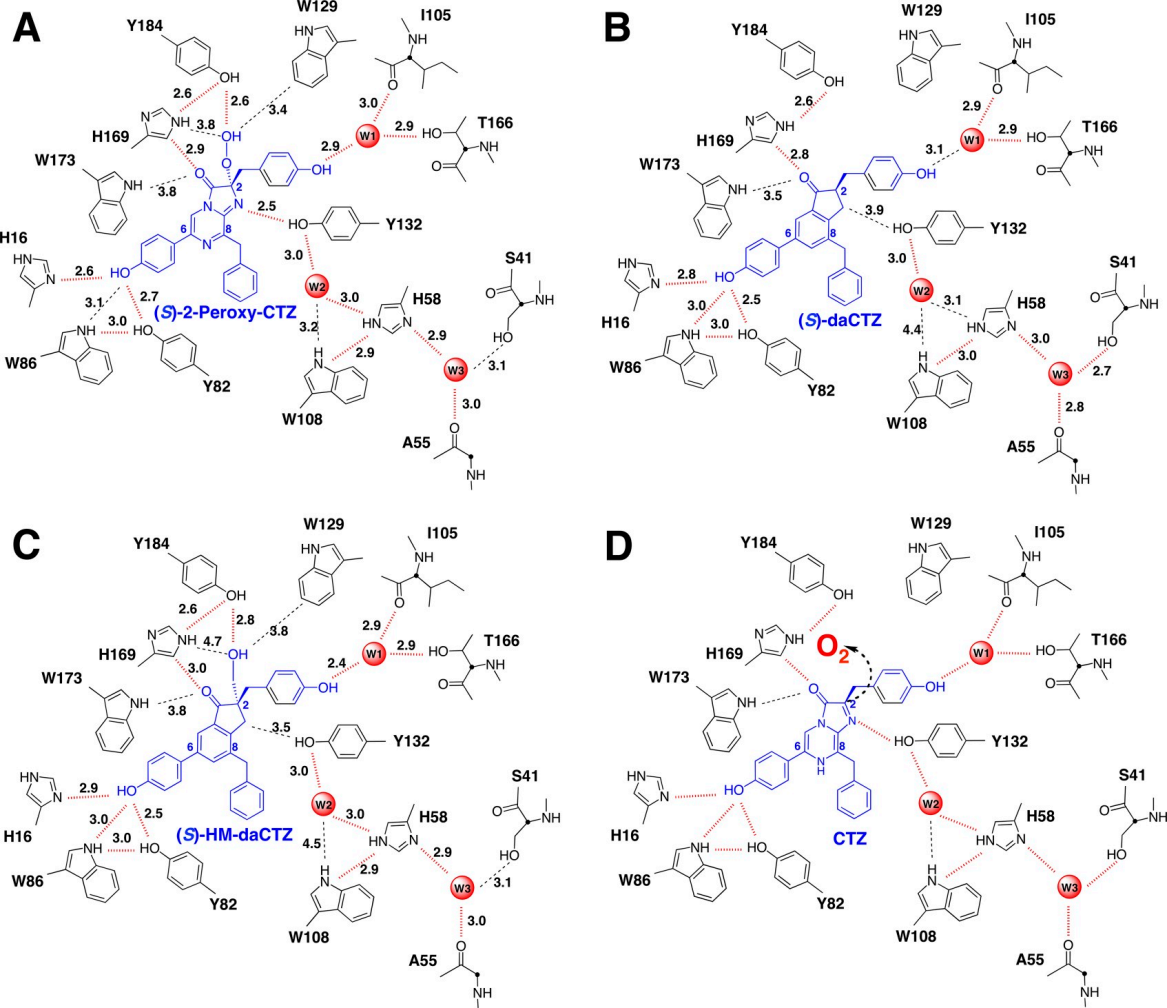
Comparison of the hydrogen-bonding networks in the (*S*)-2-peroxycoelenterazine binding cavity among native aequorin (A), the apoAequorin/(*S*)-daCTZ complex (B), the apoAequorin/(*S*)-HM-daCTZ complex (C), and the predicted binding of coelenterazine in apoAequorin (D).

The asymmetric unit of the crystal contained two molecules of the deaza-CTZ analog/apoAequorin complex and formed 16 molecules of the deaza-CTZ analog/apoAequorin complex, similar to the case of *cf3*-aequorin [20]. The molecules in the crystalline asymmetric unit were almost identical and divided into two structurally distinguishable forms “A” and “B”, observed in native aequorin (PDB ID: 1EJ3). The overall structures of the deaza-analog complexes were basically the same as that of native aequorin (Fig 8), and the schematic representations of the hydrogen-bonding networks surrounding the deaza-analogs in the apoAequorin molecule are shown in Fig 9.

In the apoAequorin/(*S*)-daCTZ complex, the *p*-hydroxy moiety at the C6-hydroxyphenyl group was stabilized by the amino acid triad of His16, Tyr82, and Trp86, and the C3-carbonyl group of the indanone structure in (*S*)-daCTZ interacted with His169 in hydrogen-bonding distances (Fig 9B). The *p*-hydroxy moiety at the C2-hydroxyphenyl group was stabilized with I105 and T166 through a water molecule, identical to that of native aequorin (Fig 9A). Previously, *h*-coelenterazine, lacking the hydroxy moiety at the C2-hydroxyphenyl group, was used as a substrate to generate active semi-synthetic *h*-aequorin [26–28]. In contrast, *bis*-coelenterazine, lacking the hydroxy moiety at both C2- and C6-hydroxyphenyl groups, could not be used for preparing active aequorin [28–30]. Thus, apoAequorin can recognize the *p*-hydroxy moiety at the C6-hydroxyphenyl group and the C3-carbonyl moiety in coelenterazine to bind coelenterazine (Fig 9D).

Native aequorin consists of apoAequorin and (*S*)-2-peroxycoelenterazine [5], and the (*S*)-2-peroxy group is stabilized by the hydrogen-bonding interaction with the hydroxy group of Tyr184 (Fig 9A). Similarly, in the apoAequorin/(*S*)-HM-daCTZ complex, the hydroxymethyl group at the C2 position of (*S*)-HM-daCTZ and the C3-carbonyl moiety were stabilized with Tyr184 and His169, respectively (Fig 9C). Also, the hydroxy moiety at the C6-hydroxyphenyl group was stabilized with His16, Tyr82, and Trp86, identical to that of native aequorin. These results suggested that the C6-hydroxyphenyl group and the C3-carbonyl group in coelenterazine are essential to bind the apoAequorin molecule (Fig 9D). Under the oxidation process of coelenterazine with O_2_ in the apoAequorin molecule, O_2_ attacks the C2-carbanion of an imidazopyrazinone ring from *Si*-face (Fig 9D) to produce (*S*)-2-peroxycoelenterazine, and it is stabilized in the apoAequorin molecule. On the other hand, the possibility of the (*R*)-2-peroxycoelenterazine formation by O_2_ attack to the C2-carbanion from *Re-*face cannot be ruled out. In this case, the conversion of the (*R*)-2-peroxy group to the (*S*)-2-peroxy form is required to be stabilized by the hydrogen-bonding interaction with the hydroxy group of Tyr184.

### Proposed mechanism of the oxidation process of coelenterazine to reconstitute the complex of (*S*)-2-peroxycoelenterazine and apoAequorin

**Fig 10:**
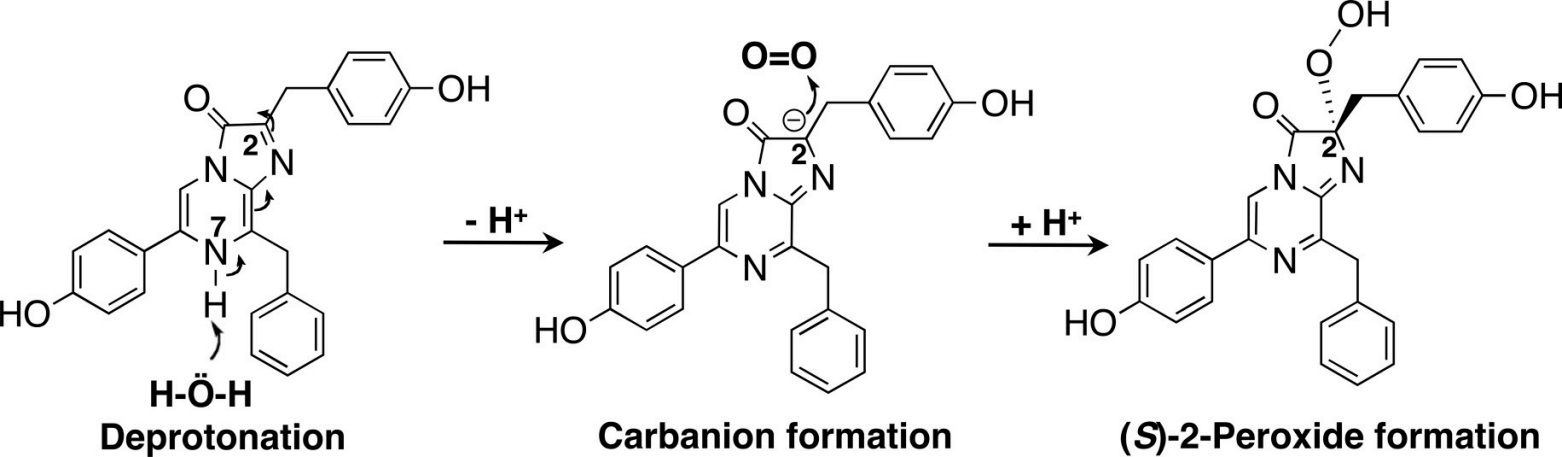
Proposed mechanism for the carbanion formation of coelenterazine in the regeneration process from apoAequorin, coelenterazine, and O_2_.

Coelenterazine is an unstable compound in aqueous solutions. For example, 2 μg of coelenterazine (1 μg/μL dissolved in ethanol, 472 pmol) in 0.1 mL of 50 mM Tris-HCl (pH 7.6) at 25°C decomposed over 50% of coelenterazine in 2 h by air oxidation. The quantities of the products were 149 pmol (coelenterazine), 68 pmol (coelenteramide), 65 pmol (coelenteramine), 90 pmol (dehydrocoelenterazine), and other unknown products (S3 Fig in S1 File). After adding apoAequorin to the regeneration mixture containing coelenterazine, coelenterazine binds to apoAequorin. This is followed by the stereospecific addition of O_2_ at the C2 position of coelenterazine to produce (*S*)-2-peroxycoelenterazine. The resultant peroxide is immediately stabilized by the hydrogen-bonding interaction with the hydroxy group of Tyr184 in apoAequorin. In the aequorin regeneration process, the carbanion formation at the C2 position of coelenterazine is probably occurring by the deprotonation at the N7 position of coelenterazine (Fig 10). Because the proton-acceptable amino acid residues such as a histidine residue in aequorin molecule were not observed at a 3.0 Å distance from the N7 position of coelenterazine, the water (H-Ö-H) was presumably acting as a base to accept a proton (Fig 10). This explanation is in good agreement with the instability of coelenterazine in aqueous solutions (S3 Fig in S1 File). The regeneration time to aequorin from apoAequorin and coelenterazine required over few hours, and this long regeneration time is probably because of the slow deprotonation and/or the slow additive reaction of O_2_ to the carbanion of coelenterazine in the apoAequorin molecule.

## Conclusion

In this study, the synthetic methods of the chiral deaza-analogs for coelenterazine and 2-peroxycoelenterazine were improved. Moreover, the deaza-analogs were used as a probe to investigate the molecular recognition of apoAequorin with coelenterazine. In the regeneration step to aequorin by incubating apoAequorin with coelenterazine, the chiral deaza-analogs of (*S*)-daCTZ and (*S*)-HM-daCTZ selectively inhibited the aequorin regeneration. The apoAequorin complexes with (*S*)-daCTZ and (*S*)-HM-daCTZ were isolated and the crystal structures of these complexes were determined. As a result, the hydroxy moiety at the C6-hydroxyphenyl group and the carbonyl moiety of the imidazopyrazinone ring in coelenterazine are crucial to bind apoAequorin molecule. Therefore, the chiral deaza-analogs of coelenterazine can be used as a probe to study the interaction of coelenterazine with proteins including photoprotein, luciferase, and coelenterazine-binding protein.

## Supporting information

S1 Methods Synthesis of *rac*-daCTZ for (*S*)- and (*R*)-daCTZ and *rac*-HM-daCTZ for (*S*)- and (*R*)-HM-daCTZ and their separation to each enantiomer. (DOC)

S1 File (PDF)

## Abbreviations

CTZ: coelenterazine
daCTZ: deaza-CTZ
HM-daCTZ: 2-hydroxymethyl-deaza-CTZ
CTMD: coelenteramide
*I*_max_: maximum intensity of luminescence
rlu: relative light units
EDTA: ethylenediaminetetraacetic acid
TE: 10 mM Tris-HCl (pH 7.6)–2 mM EDTA
