# Supplementary material for: Chiral deaza-coelenterazine analogs for probing a substrate-binding site in the Ca2+-binding photoprotein aequorin
Source: PLoS One. 2021 Jun 11;16(6):e0251743. doi: 10.1371/journal.pone.0251743 (PMC8195370; doi:10.1371/journal.pone.0251743)
Supplement: S1 Methods — (DOC) [file pone.0251743.s001.doc]

**S1 Methods**

**Synthesis of *rac*-daCTZ for (*S*)- and (*R*)-daCTZ and *rac*-HM-daCTZ for (*S*)- and (*R*)-HM-daCTZ and their separation to each enantiomer**

***General remarks***

Silica-gel column chromatography was conducted with medium pressure liquid chromatography (MPLC) (Yamazen, Smart Flash AI-580S) using a silica-gel-packed column (Biotage Zip cartridge). Recycling preparative high performance liquid chromatography (HPLC) was conducted with LC-Forte/R system (YMC Co., Ltd.) using a high-resolution gel permeation chromatography (GPC) column (YMC Co., Ltd., YMC-GPC T2000 or YMC-GPC T2000-40). Preparative HPLC was performed on JASCO HPLC Systems that consists of PU-2089Plus (liquid chromatograph), MD-2010Plus (diode array detector), and LC-NetII/ADC (communications bus) units. Analytical HPLC was performed on JASCO HPLC Systems that consists of PU-2089Plus (liquid chromatograph), MD-2010Plus (diode array detector), and LC-NetII/ADC (communication bus) units, or Shimadzu HPLC Systems that consists of LC-20AD (liquid chromatograph), DGU-20A3R (degasser), SPD-20A (UV/vis detector), and CTO-20A (column oven) units. Specific optical rotation ([α]\s\up 6( )) was measured on a JASCO P-2100 Digital Polarimeter using a 5 cm-length cell. Melting points (mp) were measured on an OptiMelt MPA100 automated melting point apparatus (Stanford Research Systems) and are uncorrected. ^1^H NMR (400 MHz) and ^13^C NMR (100 MHz) spectra obtained from measurements at ambient temperature were taken on a JEOL 400SS spectrometer. CDCl_3_ containing 0.03% tetramethylsilane (TMS) (CIL, Cat. No. DLM-7) and CD_3_OD (CIL, Cat. No. DLM-24) were used as solvents for obtaining NMR spectra. Chemical shifts (δ) are given in parts per million (ppm) downfield from TMS (δ 0.00 ppm for ^1^H NMR in CDCl_3_) or the solvent peak (δ 77.0 ppm for ^13^C NMR in CDCl_3_), or BF_3_**·**OEt_2_ (δ 0.0 ppm for ^11^B NMR in CDCl_3_) as an internal reference with coupling constants (*J*) in hertz (Hz). The abbreviations s, d, m, and br signify singlet, doublet, multiplet, and broad, respectively. IR spectra were measured by single reflection ATR method on a Thermo Scientific Nicolet iS5 FT-IR with the absorption band given in cm^−1^. High-resolution mass spectra (HRMS) were measured on a Thermo Scientific Exactive Plus Orbitrap under positive (ESI^+^) conditions.

***Experimental procedures and analytical data of compounds***

**4-Bromo-6-(4,4,5,5-tetramethyl-1,3,2-dioxaborolan-2-yl)-1-indanone (2)**

Under an argon atmosphere, a solution of 4-bromo-1-indanone (**1**) (commercial, 1.06 g, 5.02 mmol), bis(pinacolato)diboron (B_2_pin_2_) (3.81 g, 15.0 mmol), [Ir(OMe)(cod)]_2_ (33.1 mg, 49.9 μmol), and 4,4′-di-*tert*-butyl-2,2′-bipyridyl (dtbpy) (26.8 mg, 99.9 μmol) in THF (30 mL, dehydrated) put in an autoclave was stirred with heating at 120 °C (oil bath) for 15 h. After cooling to room temperature, the mixture was diluted with *n*-hexane (ca. 30 mL) and the solution was passed through a thin pad of silica-gel using *n*-hexane/EtOAc (20/1) as an eluent. The resulting solution was concentrated under reduced pressure and the residue was purified by recycling preparative HPLC (GPC, chloroform) to give 4-bromo-6-(4,4,5,5-tetramethyl-1,3,2-dioxaborolan-2-yl)-1-indanone (**2**) (1.43 g, 4.24 mmol, 84.5%) as a colorless solid. TLC *R*_f_ = 0.40 (*n*-hexane/EtOAc = 5/1); mp 133–135 ºC; ^1^H NMR (400 MHz, CDCl_3_): δ 8.17 (s, 1H), 8.16 (s, 1H), 3.13–3.07 (m, 2H), 2.76–2.70 (m, 2H), 1.35 (s, 12H); ^13^C{^1^H} NMR (100 MHz, CDCl_3_): δ 205.9, 157.4, 143.1, 138.7, 129.2, 122.1, 84.4 (2C), 36.1, 27.2, 24.8 (4C) (the signal for the carbon attached to the boron atom was not observed); ^11^B NMR (128 MHz, CDCl_3_) δ 29.1; IR (neat, cm^−1^) 2976, 2927, 1721, 1607, 1405, 1335, 1270, 1235, 967, 850, 678; HRMS (ESI^+^) *m*/*z*: [M + H]^+^ calcd for C_15_H_19_B^79^BrO_3_^+^, 337.0605; found, 337.0604.

**4-Bromo-6-(4-methoxyphenyl)-1-indanone (3)**

Under an argon atmosphere, to a solution of **2** (1.01 g, 3.00 mmol), 4-iodoanisole (2.11 g, 9.01 mmol), PdCl_2_(dppf)·CH_2_Cl_2_ (123 mg, 151 μmol), and DPPF (83.2 mg, 150 μmol) in a 1/1 mixture of toluene/EtOH (30 mL each) was added a solution of potassium carbonate (1.24 g, 8.97 mmol) in distilled water (4.5 mL) and the mixture was stirred with heating at 65 °C (oil bath) for 19.5 h. After cooling to room temperature, to the mixture was added aqueous NH_4_Cl (saturated, ca. 50 mL) and extracted with EtOAc. The combined organic extract was dried over Na_2_SO_4_, filtered, and the filtrate was concentrated under reduced pressure. The residue was purified by column chromatography (MPLC, *n*-hexane/EtOAc = 100/0 to 50/50) to afford 4-bromo-6-(4-methoxyphenyl)-1-indanone (**3**) (735 mg, 2.32 mmol, 77.2%) as a colorless solid. TLC *R*_f_ = 0.35 (*n*-hexane/EtOAc = 5/1); mp 98–100 ºC; ^1^H NMR (400 MHz, CDCl_3_): δ 7.98 (s, 1H), 7.88 (s, 1H), 7.53 (d, *J* = 9.0 Hz, 2H), 6.99 (d, *J* = 9.0 Hz, 2H), 3.86 (s, 3H), 3.13–3.07 (m, 2H), 2.82–2.75 (m, 2H); ^13^C{^1^H} NMR (100 MHz, CDCl_3_): δ 206.2, 159.8, 152.7, 142.5, 139.4, 135.7, 131.1, 128.2 (2C), 122.4, 120.2, 114.5 (2C), 55.4, 36.6, 26.6; IR (neat, cm^−1^) 2916, 2833, 1721, 1608, 1516, 1458, 1319, 1179, 1039, 850, 793; HRMS (ESI^+^) *m*/*z*: [M + Na]^+^ calcd for C_16_H_13_^79^BrO_2_Na^+^, 340.9971; found, 340.9967.

**4-Benzyl-6-(4-methoxyphenyl)-1-indanone (4)**^1,2^

Under an argon atmosphere, a solution of **3** (1.20 g, 3.78 mmol), potassium benzyltrifluoroborate (2.25 g, 11.4 mmol), PdCl_2_(dppf)·CH_2_Cl_2_ (309 mg, 378 μmol), and cesium carbonate (4.93 g, 15.1 mmol) in THF (30 mL) and distilled water (3 mL) was stirred with heating at 70 °C (oil bath) for 24 h. After cooling to room temperature, to the mixture was added aqueous NH_4_Cl (saturated, ca. 30 mL) and extracted with EtOAc. The combined organic extract was dried over Na_2_SO_4_, filtered, and the filtrate was concentrated under reduced pressure. The residue was purified by column chromatography (MPLC, *n*-hexane/EtOAc = 100/0 to 50/50) to afford 4-benzyl-6-(4-methoxyphenyl)-1-indanone (**4**) (1.20 g, 3.65 mmol, 96.2%) as a colorless amorphous. TLC *R*_f_ = 0.40 (*n*-hexane/EtOAc = 3/1); ^1^H NMR (400 MHz, CDCl_3_): δ 7.83 (d, *J* = 2.0 Hz, 1H), 7.63 (d, *J* = 2.0 Hz, 1H), 7.55–7.50 (AA’BB’, 2H), 7.34–7.28 (m, 2H), 7.24–7.18 (m, 3H), 7.01–6.95 (AA’BB’, 2H), 4.11 (s, 2H), 3.81 (s, 3H), 3.05–2.97 (m, 2H), 2.73–2.69 (m, 2H); ^13^C{^1^H} NMR (100 MHz, CDCl_3_): δ 207.3, 159.4, 152.6, 140.7, 139.1, 139.0, 138.0, 133.9, 132.5, 128.8 (2C), 128.7 (2C), 128.2 (2C), 126.4, 119.5, 114.3 (2C), 55.4, 38.6, 36.6, 24.4; IR (neat, cm^−1^) 2929, 1704, 1607, 1515, 1429, 1286, 1246, 1175, 1031, 841, 747, 700; HRMS (ESI^+^) *m*/*z*: [M + Na]^+^ calcd for C_23_H_20_O_2_Na^+^, 351.1356; found, 351.1351.

**(*E*)-4-Benzyl-2-(4-methoxybenzylidene)-6-(4-methoxyphenyl)-1-indanone (5)**^1,2^

Under an argon atmosphere, to a solution of **4** (209 mg, 0.638 mmol) in EtOH (5 ml) were added aqueous sodium hydroxide solution (2.5 M, 357 μL, 0.893 mmol) and *p*-anisaldehyde (100 μL, 0.829 mmol). After stirring the mixture with heating at 50 ºC for 21.5 h, the resultant yellow suspension was cooled to room temperature. The solid was collected by filtration using a Kiriyama funnel, washed with ether (5 mL), and dried under reduced pressure to afford (*E*)-4-benzyl-2-(4-methoxybenzylidene)-6-(4-methoxy phenyl)-1-indanone (**5**) (256 mg, 0.570 mmol, 89.7%) as a pale yellow solid. TLC *R*_f_ = 0.25 (*n*-hexane/EtOAc = 2.5/1); mp 198–200 ºC; ^1^H NMR (400 MHz, CDCl_3_): δ 7.98 (d, *J* = 1.5 Hz, 1H), 7.66–7.58 (m, 4H), 7.57–7.52 (AA’BB’, 2H), 7.36–7.31 (m, 2H), 7.26–7.22 (m, 3H), 7.01–6.95 (m, 4H), 4.20 (s, 2H), 3.87 (s, 3H), 3.85 (s, 3H), 3.84 (m, 2H); ^13^C{^1^H} NMR (100 MHz, CDCl_3_): δ 194.6, 160.8, 159.4, 140.9, 139.2, 139.1, 138.2, 133.8, 133.6, 132.7, 132.6 (2C), 128.8 (2C), 128.7 (2C), 128.2 (2C), 128.1, 126.5 (2C), 120.1 (2C), 114.5 (2C), 114.3 (2C), 55.4, 55.3, 38.6, 31.0; IR (neat, cm^−1^) 2829, 1683, 1599, 1510, 1454, 1439, 1250, 1175, 1032, 760, 707, 545; HRMS (ESI^+^) *m*/*z*: [M + H]^+^ calcd for C_31_H_27_O_3_^+^, 447.1955; found, 447.1951.

**4-Benzyl-2-(4-methoxybenzyl)-6-(4-methoxyphenyl)-1-indanone (6)**^1,2^

Under an argon atmosphere, to a suspension of Pd(OAc)_2_ (5.6 mg, 25 μmol), PCy_3_ (14 mg, 50 μmol), and (*E*)-4-benzyl-2-(4-methoxybenzylidene)-6-(4-methoxyphenyl)-1-indanone (**5**) (223 mg, 0.500 mmol) in toluene (10 mL, dehydrated) was added (EtO)_3_SiH (138 μL, 0.750 μmol) and the mixture was stirred with heating at 90 ºC (oil bath) for 19 h. After cooling to room temperature, to the mixture was added TBAF (1 M in THF, 2 mL, 2 mmol) and stirred for 2 h at the same temperature. To the mixture was added water (ca. 10 mL) and extracted with EtOAc (ca. 10 mL × 3). The combined organic extract was dried over Na_2_SO_4_, and after filtration, the filtrate was concentrated under reduced pressure. The residue was purified by silica-gel column chromatography (MPLC, *n*-hexane/EtOAc = 100/0 to 50/50) to afford 4-benzyl-2-(4-methoxybenzyl)-6-(4-methoxyphenyl)-1-indanone (**6**) (225 mg, 0.502 mmol, quant) as a yellow solid. TLC *R*_f_ = 0.30 (*n*-hexane/EtOAc = 5/1);

mp 128–130 ºC; ^1^H NMR (400 MHz, CDCl_3_): δ 7.83 (d, *J* = 2.0 Hz, 1H), 7.58 (d, *J* = 2.0 Hz, 1H), 7.53–7.49 (AA’BB’, 2H), 7.35–7.28 (m, 2H), 7.24–7.11 (m, 3H), 7.10–7.07 (AA’BB’, 2H), 6.98–6.95 (AA’BB’, 2H), 6.84–6.80 (AA’BB’, 2H), 4.03 (s, 2H), 3.84 (s, 3H), 3.79 (s, 3H), 3.28 (dd, *J* = 5.0, 17.5 Hz, 1H), 3.07–2.94 (m, 2H), 2.72–2.59 (m, 2H);

^13^C{^1^H} NMR (100 MHz, CDCl_3_): δ 208.3, 159.4, 158.1, 151.1, 140.8, 139.1, 138.9, 137.4, 134.0, 132.5, 131.4, 129.9 (2C), 128.8 (2C), 128.6 (2C), 128.2 (2C), 126.4, 119.7, 114.3 (2C), 113.9 (2C), 55.4, 55.2, 49.4, 38.4, 36.3, 30.4; IR (neat, cm^−1^) 2924, 1698, 1509, 1466, 1241, 1174, 1050, 810, 796, 700, 578; HRMS (ESI^+^) *m*/*z*: [M + Na]^+^ calcd for C_31_H_28_NaO_3_^+^, 471.1931; found, 471.1930.

**4-Benzyl-2-(4-hydroxybenzyl)-6-(4-hydroxyphenyl)-1-indanone** (***rac*-daCTZ, *rac*-7**)^1,2^

Under an argon atmosphere, to a solution of 4-benzyl-2-(4-methoxybenzyl)-6-(4-methoxyphenyl)-1-indanone (**6**) (224 mg, 0.500 mmol) in dichloromethane (10 mL, dehydrated) was added boron tribromide (1 M in dichloromethane, 2 mL, 2 mmol) at −78 ºC. After warming up to room temperature, the mixture was stirred for 8 h. The reaction was quenched with ice and the resulting mixture was extracted with dichloromethane (ca. 10 mL × 3). The organic layer was dried over Na_2_SO_4_, and after filtration, the filtrate was concentrated under reduced pressure. The residue was purified by silica-gel column chromatography (MPLC, *n*-hexane/EtOAc = 95/5 to 5/95) followed by recycling preparative HPLC (GPC, chloroform) to afford 4-benzyl-2-(4-hydroxybenzyl)-6-(4-hydroxyphenyl)-1-indanone (*rac*-daCTZ, *rac*-**7**) (145 mg, 0.345 mmol, 69.0%) as a pale brown solid. TLC *R*_f_ = 0.45 (*n*-hexane/EtOAc = 1/1);

mp 102–104 ºC; ^1^H NMR (400 MHz, CDCl_3_): δ 7.82 (d, *J* = 2.0 Hz, 1H), 7.58 (d, *J* = 2.0 Hz, 1H), 7.47–7.43 (AA’BB’, 2H), 7.31–7.27 (m, 2H), 7.24–7.11 (m, 3H), 7.04–7.02 (AA’BB’, 2H), 6.92–6.88 (AA’BB’, 2H), 6.76–6.72 (AA’BB’, 2H), 5.04 (br, 1H), 4.74 (br, 1H), 4.03 (s, 2H), 3.25 (dd, *J* = 5.0, 17.5 Hz, 1H), 3.07–2.93 (m, 2H), 2.72–2.59 (m, 2H); ^13^C{^1^H} NMR (100 MHz, CDCl_3_): δ 208.5, 155.5, 154.0, 151.2, 140.8, 139.1, 138.9, 137.4, 134.1, 132.7, 131.5, 130.1 (2C), 128.7 (2C), 128.6 (2C), 128.4 (2C), 126.4, 119.7, 115.8 (2C), 115.3 (2C), 49.4, 38.4, 36.3, 30.4; IR (neat, cm^−1^) 3316, 3024, 2920, 1686, 1610, 1595, 1513, 1474, 1224, 1172, 1064, 831, 736, 531, 514; HRMS (ESI^+^) *m*/*z*: [M + Na]^+^ calcd for C_29_H_24_NaO_3_^+^, 443.1618; found, 443.1617.

**(2*R*)-4-Benzyl-2-(4-hydroxybenzyl)-6-(4-hydroxyphenyl)-1-indanone ((*R*)-daCTZ, (*R*)-7) and (2*S*)-4-benzyl-2-(4-hydroxybenzyl)-6-(4-hydroxyphenyl)-1-indanone ((*S*)-daCTZ, (*S*)-7)^1,2^**

A part of the racemic mixture *rac*-**7** (*rac*-daCTZ) was separated by preparative MPLC (DAICEL CHIRALFLASH IC (30 mm i.d. × 100 mm), *n*-hexane/chloroform/MeOH = 40/58/2, flow rate 0.28 mL/min) to give (*S*)-**7** ((*S*)-daCTZ) (*t* = 57.0 min, [α]Combin = +121.2 (*c* 0.10 in EtOH)) and (*R*)-**7** ((*R*)-daCTZ) (*t* = 82.7 min, [α]Combin = −118.6 (*c* 0.10 in EtOH). Analytical HPLC (DAICEL CHIRALPAK IC (4.6 mm i.d. × 250 mm), *n*-hexane/chloroform/MeOH = 40/58/2, flow rate = 1.5 mL/min): (*S*)-**7** (*t* = 12.1 min, 99.6% ee) and (*R*)-**7** (*t* = 17.8 min, 99.2% ee). Absolute configuration of each enantiomer was confirmed based on the reported retention time of each enantiomer on analytical HPLC (DAICEL CHIRALCEL OC-H (4.6 mm i.d. × 250 mm) connected with guard column (DAICEL CHIRALCEL OC-H, 4.0 mm i.d. × 10 mm), *n*-hexane/EtOH = 67/33, flow rate = 0.5 mL/min): (*S*)-**7** (*t* = 17.5 min) and (*R*)-**7** (*t* = 20.0 min); reported analytical HPLC (DAICEL CHIRALCEL OC (4.6 mm i.d. × 250 mm), hexane/EtOH = 2/1, flow rate 0.5 mL/min): (*S*)-**7** (*t* = 20 min) and (*R*)-**7** (*t* = 24 min).^1,2^

**4-Benzyl-2-(4-hydroxybenzyl)-2-(hydroxymethyl)-6-(4-hydroxyphenyl)-1-indanone (*rac*-HM-daCTZ, *rac*-8)**^1,2^

Under an argon atmosphere, to a solution of 4-benzyl-2-(4-hydroxybenzyl)-6-(4-hydroxyphenyl)-1-indanone (*rac*-daCTZ, *rac*-**7**) (92.0 mg, 0.219 mmol) in EtOH (10 mL) was added aqueous NaOH (2.5 M, 0.44 mL, 1.1 mmol) and formalin (36%, 92 μL, 1.1 mmol) at room temperature. After stirred for 1 h, the reaction mixture was quenched with aqueous NH_4_Cl (saturated, 10 mL) and removed EtOH under reduced pressure followed by extraction with 20/1 of dichloromethane and MeOH (ca. 10 mL × 3). The organic layer was dried over Na_2_SO_4_, and after filtration, the filtrate was concentrated under reduced pressure. The residue was purified by silica-gel column chromatography (MPLC, chloroform/MeOH = 100/0 to 80/20) to afford 4-benzyl-2-(4-hydroxybenzyl)-2-(hydroxymethyl)-6-(4-hydroxyphenyl)-1-indanone (*rac*-HM-daCTZ, *rac*-**8**) (76.7 mg, 0.350 mmol, 77.7%) as a pale brown solid.

TLC *R*_f_ = 0.50 (dichloromethane/MeOH = 10/1); mp 95–97 ºC; IR (neat, cm^−1^) 3316, 3024, 2920, 1686, 1610, 1595, 1513, 1474, 1448, 1326, 1224, 1172, 1064, 831, 703, 514;

^1^H NMR (400 MHz, CDCl_3_/CD_3_OD = 10/1): δ 7.79 (s, 1H), 7.59 (d, *J* = 2.0 Hz, 1H), 7.45–7.42 (AA’BB’, 2H), 7.36 (s, 1H), 7.31–7.27 (m, 2H), 7.24–7.11 (m, 3H), 7.02–6.89 (AA’BB’, 2H), 6.92–6.88 (AA’BB’, 2H), 6.76–6.72 (AA’BB’, 2H), 4.03 (s, 2H), 3.25–3.19 (m, 1H), 3.07–2.96 (m, 2H), 2.73–2.56 (m, 2H) (the peaks for three hydroxy protons were not observed); ^13^C{^1^H} NMR (100 MHz, CDCl_3_/CD_3_OD = 10/1): δ 209.4, 156.6, 155.0, 151.2, 141.0, 139.0, 138.8, 137.0, 134.0, 132.7, 131.3, 130.1, 129.8 (2C), 128.6 (2C), 128.4 (2C), 128.1 (2C), 126.2, 119.3, 115.6 (2C), 115.1 (2C), 38.2, 36.3, 30.2, 29.5; HRMS (ESI^+^) *m*/*z*: [M + Na]^+^ calcd for C_30_H_26_O_4_^+^, 473.1723; found, 473.1726.

**(2*R*)-4-Benzyl-2-(4-hydroxybenzyl)-2-(hydroxymethyl)-6-(4-hydroxyphenyl)-1-indanone ((*R*)-HM-daCTZ, (*R*)-8) and (2*S*)-4-benzyl-2-(4-hydroxybenzyl)-2-(hydroxymethyl)-6-(4-hydroxyphenyl)-1-indanone ((*S*)-HM-daCTZ, (*S*)-8)**^1,2^

A part of the racemic mixture *rac*-**8** (*rac*-HM-daCTZ) was separated by preparative HPLC (DAICEL CHIRACEL OJ (10 mm i.d. × 250 mm) connected with guard column (DAICEL CHIRALCEL OJ, 10 mm i.d. × 50 mm), *n*-hexane/EtOH = 40/60 to 10/90, flow rate 3.0 mL/min) to give (*R*)-**8** ((*R*)-HM-daCTZ) (*t* = 16.4 min, [α]Combin = −87.4 (*c* 1.0 in EtOH)) and (*S*)-**8** ((*S*)-HM-daCTZ) (*t* = 26.0 min, [α]Combin = +90.7 (*c* 1.0 in EtOH)). Analytical HPLC (DAICEL CHIRALCEL OJ-3 (4.6 mm i.d. × 250 mm) connected with guard column (DAICEL CHIRALCEL OJ-3, 4.0 mm i.d. × 10 mm), *n*-hexane/EtOH = 50/50, flow rate = 0.5 mL/min): (*R*)-**8** (*t* = 22.5 min, 97.3% ee) and (*S*)-**8** (*t* = 32.3 min, 97.0% ee). The slight decrease of enantiomeric excess was observed for both enantiomers. This is probably because HM-daCTZ is easy to cause racemization via the retro-aldol reaction. Immediate use after the HPLC separation and/or careful storage is recommended for HM-daCTZ. Absolute configuration of each enantiomer was confirmed based on the reported specific optical rotation: (*R*)-**8**: [α]Combin = −107 (*c* 0.02 in EtOH) and (*S*)-**8**: [α]Combin = +110 (*c* 0.02 in EtOH).^2^ The peaks of enantiomers appeared in the same order on analytical HPLC; DAICEL CHIRALCEL OC (4.6 mm i.d. × 250 mm), *n*-hexane/EtOH = 6/1, flow rate = 0.5 mL/min): (*S*)-**8** (*t* = 106.6 min) and (*R*)-**8** (*t* = 113.1 min) with that reported; DAICEL CHIRALCEL OC (4.6 mm i.d. × 250 mm), hexane/EtOH = 6/1, flow rate 0.5 mL/min): (*S*)-**8** (*t* = 33 min) and (*R*)-**8** (*t* = 38 min).^2^ Although we conducted the analyses under the same conditions using the same type of column, both enantiomers showed significantly longer retention time compared with the reported values.

**References**

1. Nakamura, H.; Wu, C.; Inouye, S.; Murai, A. *J. Am. Chem. Soc.* **2001**, *123*, 1523–1524.

2. Wu, C.; Nakamura, H.; Murai, A.; Inouye, S. *Tetrahedron* **2001**, *57*, 9575–9583.
