# Supplementary material for: Chiral deaza-coelenterazine analogs for probing a substrate-binding site in the Ca2+-binding photoprotein aequorin
Source: PLoS One. 2021 Jun 11;16(6):e0251743. doi: 10.1371/journal.pone.0251743 (PMC8195370; doi:10.1371/journal.pone.0251743)
Supplement: S1 File — (PDF) [file pone.0251743.s002.pdf]

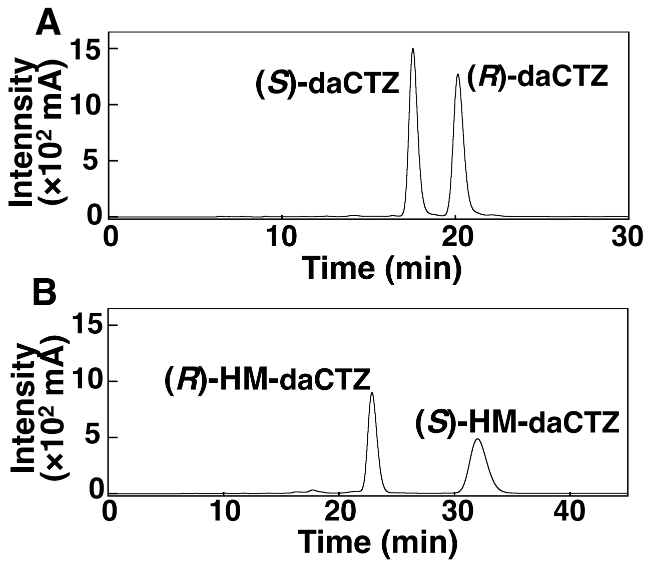

S1 Fig.

HPLC analyses of *rac*-daCTZ ((*S*)- and (*R*)-daCTZ) and *rac*-HM-daCTZ ((*S*)- and (*R*)-HM-daCTZ) using a chiral column

(A) Analysis of *rac*-daCTZ for (*S*)-daCTZ and (*R*)-daCTZ using a CHIRALCEL OC-H column (4.6 × 250 mm, Daicel Corp.) with a guard cartridge (4 × 10 mm) in a mobile phase of *n*-hexane/ethanol (2:1). Sample: *rac*-daCTZ (17 μg) dissolved in 20 μL *n*-hexane/ethanol (2:1).

(B) Analysis of *rac*-HM-daCTZ for (*S*)-HM-daCTZ and (*R*)-HM-daCTZ using a CHIRALCEL column OJ-3 (4.6 × 250 mm, Daicel Corp.) with a guard cartridge (4 × 10 mm) in a mobile phase of *n*-hexane/ethanol (1:1). Sample: *rac*-HM-daCTZ (18 μg) dissolved in 20 μL of *n*-hexane/ethanol (1:1).

The sample was analyzed using Shimadzu HPLC system consisting of LC-20AD (pump), DGU-20A3R (degassing unit), SPD-20A (UV-VIS detector), and CTO-30A (column oven). HPLC conditions: flow rate, 0.5 mL/min; column temp, 40 °C ; detector, 254 nm; sample injection, 20 μL.

A. apoAequorin/(*S*)-HM-daCTZ complex

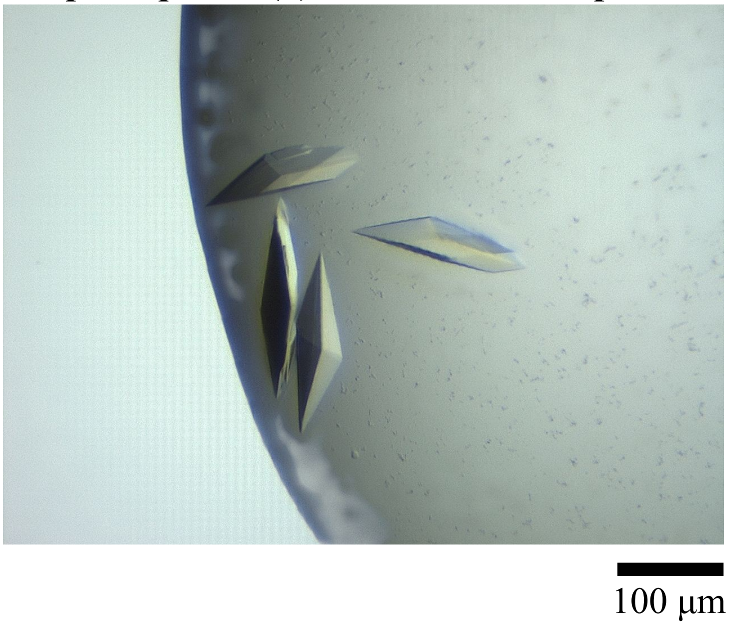

B. apoAequorin/(*S*)-daCTZ complex

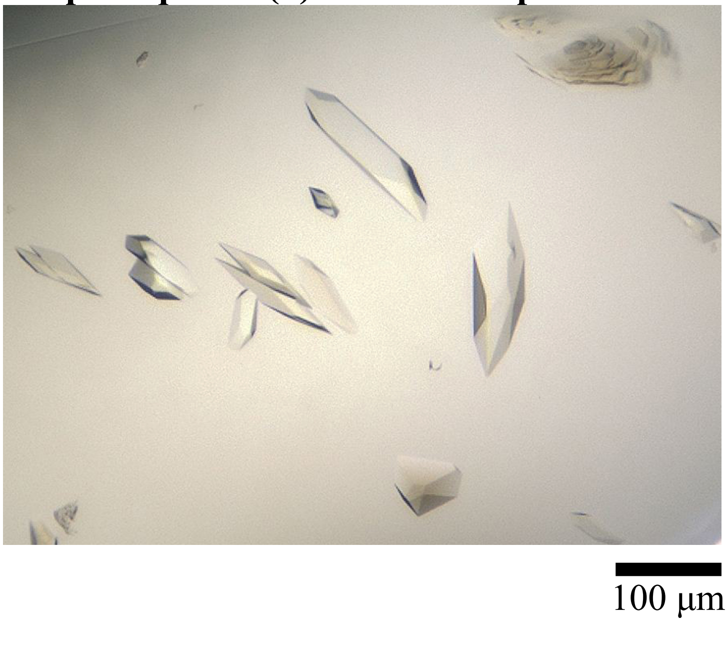

S2 Fig.

Photographs of the apoAequorin/(*S*)-HM-daCTZ complex (A) and the apoAequorin/(*S*)-daCTZ complex (B)

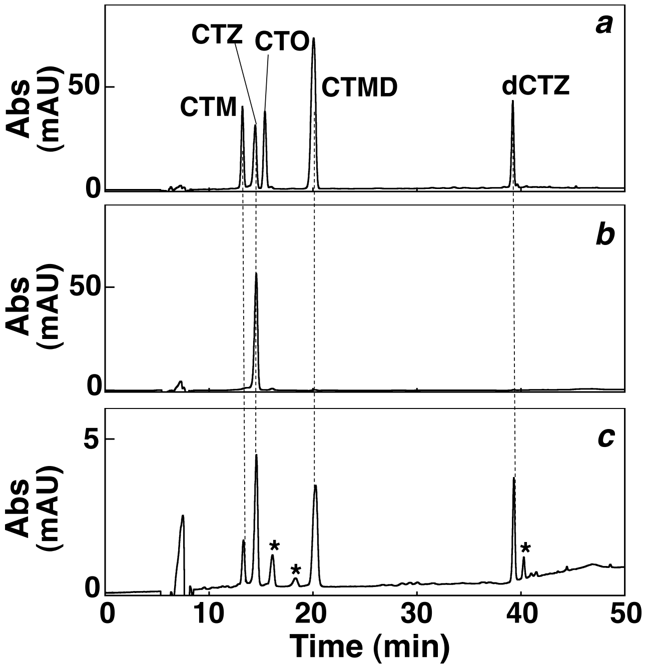

S3 Fig.

HPLC analysis of degradation products from coelenterazine incubated in 50 mM Tris-HCl (pH 7.6) at 25 °C for 2 h

Abbreviations are as follows: coelenteramine (CTM), coelenterazine (CTZ), 3-benzyl-5-(4-hydroxyphenyl)pyrazin-2(1*H*)-one (CTO), coelenteramide (CTMD), dehydrocoelenterazine (dCTZ), and unknown products (*).

a. Authentic samples of CTM, CTZ, CTO, CTMD, and dCTZ (0.5 µg each, in 10 µL of ethanol).

b. Coelenterazine (1 µg in 10 µL of ethanol) used in this experiment.

c. The reaction mixtures contain 2 µg of coelenterazine (1 µg/µL in ethanol) in 50 mM Tris-HCl (pH 7.6) and incubate at 25 °C for 2 h.

The reaction mixtures were extracted with 500 µL of ethyl acetate by mixing using a vortex mixer for 30 sec, centrifuged at 10,000 rpm for 3 min, and the ethyl acetate layer (450 µL) was recovered, dry down *in vacuo*, dissolved in 100 µL of ethanol, and 10 µL of sample solution was subjected to an Agilent (CA, USA) 1200 series HPLC system. HPLC conditions were as follows: column, Wakosil 5C4 (5 mm, 4.6 mm × 250 mm, Column No. 05413); eluting solvent, CH_3CN containing 0.1% trifluoroacetic acid; gradient elution, 40% CH_3CN for 10 min, 40–50% CH_3CN for 20 min, 50–80% CH_3CN for 10 min, and 80% CH_3CN for 10 min; flow rate, 0.5 mL/min; and detector, 330 nm.
